# Among 69,178 UK Residents Ages 65+ Years, Frailty Associates Significantly With Lifestyle Behaviors and Depression: A Cross‐Sectional Study

**DOI:** 10.1002/hsr2.70593

**Published:** 2025-03-26

**Authors:** Jisu Kim, Jonathan Kenyon, Juan Lu, Lana Sargent, Youngdeok Kim

**Affiliations:** ^1^ Department of Kinesiology and Health Sciences Virginia Commonwealth University Richmond Virginia USA; ^2^ Department of Epidemiology, School of Public Health Virginia Commonwealth University Richmond Virginia USA; ^3^ Department of Adult Health and Nursing System, School of Nursing Virginia Commonwealth University Richmond Virginia USA

**Keywords:** depression, frailty, lifestyle behaviors, sleep

## Abstract

**Background:**

Frailty and depression are highly prevalent in older adults. However, the complex association of their interlinked factors, including lifestyle behaviors, remains unexplored in population‐based epidemiological studies.

**Aims:**

This study aimed to evaluate the relationship between frailty and depression and to determine associated modifiable lifestyle risk factors for depression among older adults in the UK Biobank (UKB).

**Methods:**

Data were obtained from 69,178 older adults (aged ≥ 65 yrs) in the baseline survey of the UKB. Frailty status was measured using the frailty phenotype criteria (range: 0–5). Participants were classified into frail (≥ 3), pre‐frail (1–2), and non‐frail (0) groups. The outcome of depression was defined by participants who had sought medical attention for nerves, anxiety, tension, or depression. Lifestyle behaviors included the self‐reported time spent in moderate‐to‐vigorous physical activity (MVPA), screen‐based sedentary behavior, and sleep. The association of interest is examined using multivariable logistic regression models.

**Results:**

29% of participants had depression, of which 7% and 45% were frail and pre‐frail, respectively. Significant correlations are observed between frailty and lifestyle behaviors (*p's* < 0.05). Frailty is significantly associated with increased odds of having depression (Frail: aOR = 1.87, 95% CI = 1.72, 2.04); Pre‐frail: aOR = 1.22, 95% CI = 1.18, 1.27), and sleep (7–8 h/d) is associated with lower odds of having depression (aOR = 0.81, 95% CI = 0.78, 0.84).

**Conclusion:**

Frail and pre‐frail older adults have a higher likelihood of experiencing depression than their non‐frail counterparts. Meeting the recommended sleep duration (7–8 h/d) can be used as a modifiable behavioral strategy to manage or prevent depression. Further longitudinal studies are warranted to examine the time sequence in this relationship.

## 1 Introduction

Frailty is a highly prevalent clinical syndrome in older adults characterized by a decline in physiological systems due to reduced resilience and resistance to stressors [1]. A growing body of literature demonstrates a strong relationship between frailty and an elevated risk of adverse health outcomes, including mental health issues [2, 3]. The global prevalence of frailty among individuals ages 50 years and older across 62 countries was 12%, followed by 46% being prefrail [4], indicating more than half of people worldwide experience some degree of frailty or are in early stage of frailty. Similarly, the prevalence of frailty and pre‐frailty among older adults aged over 65 years in the United States was 15% and 45%, respectively [5]. Moreover, frail older adults in this cohort experienced chronic conditions (e.g., diabetes, heart disease, lung disease) and disabilities that were twice as high as those of their non‐frail counterparts [5]. With the rapidly growing aging population, frailty has become a prominent public health concern; therefore, exploring its association with other related health outcomes and modifiable risk factors is essential to reduce the health burden and improve well‐being in older adults.

Depression is a common psychiatric disorder associated with frailty in older adults, accompanied by impaired functioning, increased utilization of medical services, and low quality of life [6]. Globally, the prevalence of depression in older adults is 28.4% [7] and has been strongly associated with increased risk of morbidity, including frailty, and mortality [8, 9]. Depression and frailty often coexist among older adults, leading to compounded health consequences, such as hastened cognitive decline, greater disability, and mortality [9, 10]. Indeed, a recent meta‐analysis found that the overall prevalence of depression in individuals with frailty was 38.6%, and the prevalence of frailty in individuals with depression was 40.4%, highlighting their strong interrelationship [3]. Several studies have suggested that the high comorbidity rate of frailty and depression can be characterized as an overlapping syndrome, as they share common diagnostic criteria or similar presenting symptoms, such as low levels of physical activity (PA) or feeling exhaustion [3]. Although frailty and depression have commonalities, evidence demonstrates they are distinct constructs, and their overlapping syndrome cannot fully explain their constructs [11]. Despite challenges in disentangling their interrelationships, evidence of their high prevalence and frequent co‐occurrence in older adults suggests a critical need to explore their links more comprehensively using large‐scale population‐based data.

Lifestyle patterns can be altered with aging, and these changes can play a significant role in the development or progression of disease. Physical inactivity, increased sedentary time (ST), and disrupted sleep duration are known as risk factors for frailty [12, 13] and depression [14] among older adults. It is well‐established that sufficient PA has numerous benefits, including reduced frailty, improved physical fitness [15], and lowered risk of depression [16]. Despite the distinct effectiveness of regular PA in health, physical inactivity and ST have been increasing over time, particularly in older adults [17]. Additionally, several studies have found that both insufficient and excessive durations of sleep are associated with a high risk of depression in older adults [18]. Given these unhealthy lifestyle factors are considered risk factors for both frailty and depression, this evidence suggests the possibility of the moderating role of lifestyle behaviors in the links between frailty and depression in older adults. However, the comprehensive relationships between frailty, depression, and lifestyle behaviors, as well as the potential moderating role of lifestyle behaviors, have yet to be systematically examined in large population‐based epidemiological studies of older adults.

Therefore, this study aimed (1) to examine the associations between frailty, lifestyle behaviors, and depression (2) to explore the potential moderating role of lifestyle behaviors, particularly PA, ST, and sleep, in the relationship between frailty and depression among older adults using the large population‐based UK Biobank (UKB) data.

## 2 Methods

### 2.1 Study Design and Sample

The UKB is a large‐scale population‐based cohort study that recruited half a million participants aged 40–69 across the United Kingdom (UK) between 2006 and 2010. This study is a cross‐sectional study design using a baseline survey of UKB. Participants were initially recruited by postal invitation at baseline (5.5% response rate) and attended one of 22 UK assessment centers across England, Scotland, and Wales. Every participant underwent a touchscreen questionnaire, a nurse‐led interview, and physical measurements during the baseline assessment, and all participants provided informed consent. A detailed description of the UKB database has been presented on the UKB website. The UKB project was approved by the North West Multicenter Research Ethical Committee (11/NW/0382).

**Figure 1:**
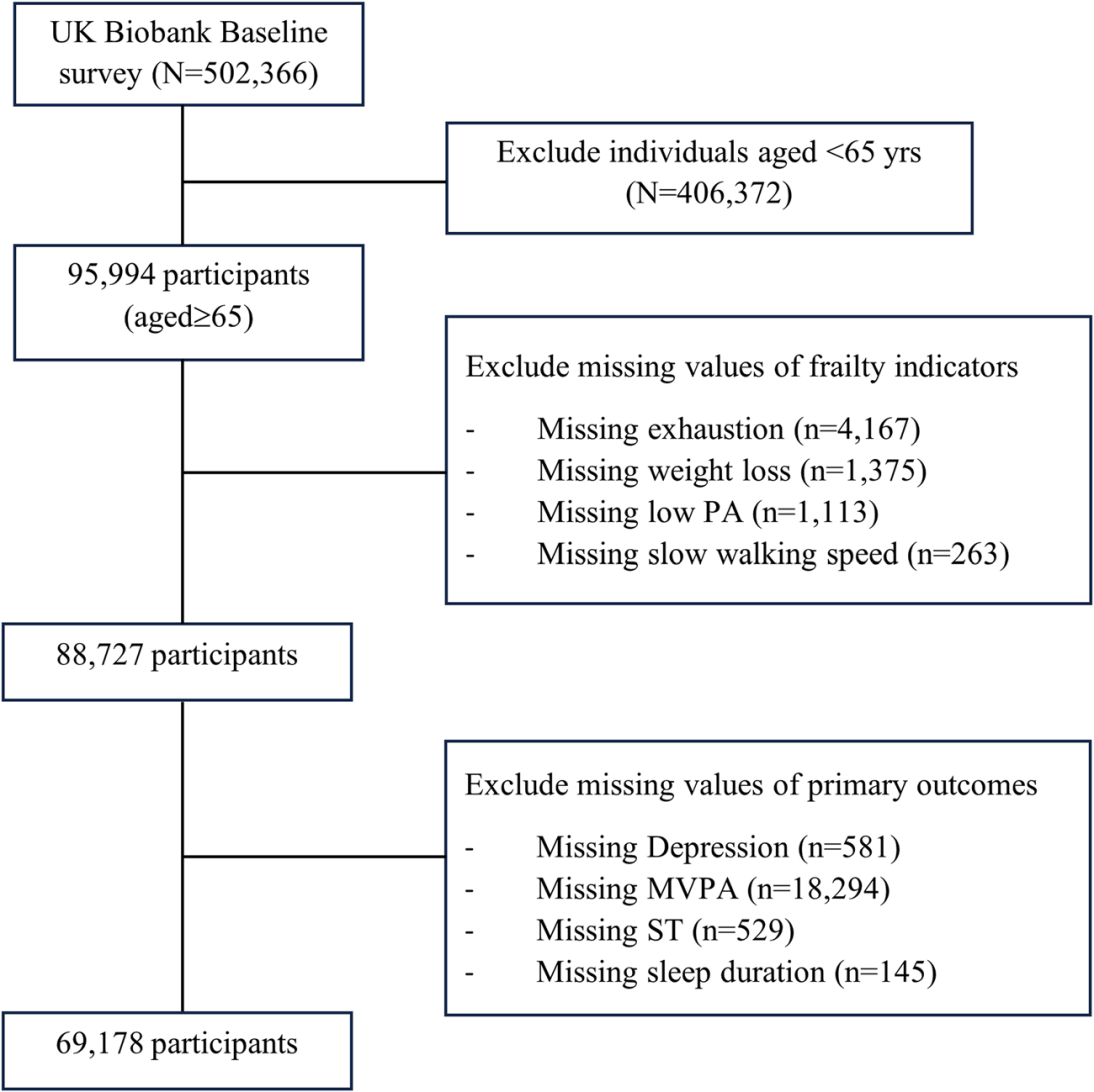
Flow diagram for study participants.

Out of 502,366 participants at baseline, 406,372 participants aged ≤ 65 years were excluded based on age criteria for the frailty phenotype [1]. Participants with missing/invalid values of any primary outcome variables were excluded. Therefore, 69,178 older adults aged ≥ 65 years were included in the current study [Figure 1].

### 2.2 Measures

#### 2.2.1 Frailty

This study utilized an adapted version of frailty phenotype criteria, initially proposed by Fried et al. in the Cardiovascular Health Study [1], tailored for the UKB developed in the previous research [2]. Frailty status was evaluated using the five frailty indicators: Exhaustion, Weight loss, Low PA (i.e., light Do It Yourself (DIY) activities – pruning, watering the lawn, etc. or none), Slow walking speed, and Low grip strength. These adaptations were made using a mixture of self‐reported and measured phenotypes that had already been validated [19]. A detailed description of the adapted definition of frailty criteria for the UKB is presented in Supporting Information: Supplementary Table 1.

Participants were classified into three different frailty groups based on the number of indicators met according to the frailty criteria [1]: frail (≥ 3 indicators), pre‐frail (1–2 indicators), and non‐frail (no indicators).

#### 2.2.2 Depression

The primary outcome of depression is defined using a broad definition developed by Howard et al. within the UK Biobank cohort [20]. The broad definition of depression has been validated in a large genome‐wide study through comparison with other diagnostic methods and international classification of disease (ICD) classification, offering a practical approach for capturing inclusive depression‐related traits [20]. Participants who had seen either a (1) general practitioner or (2) psychiatrist for nerves, anxiety, tension, or depression were defined as having depression.

#### 2.2.3 Lifestyle Behaviors

##### 2.2.3.1 Moderate‐to‐Vigorous Physical Activity

Participants' MVPA levels were determined by whether participants met the 2017 UK physical activity guidelines of 150 min of moderate‐intensity activities (e.g., brisk walking for at least 30 min 5 days/week) or 75 min of vigorous‐intensity activities per week (e.g., heavy lifting, running, fast bicycling, etc.). Participants' metabolic equivalent task (MET) minutes per week for moderate‐intensity and vigorous‐intensity PA was calculated using the international physical activity questionnaire (IPAQ) MET scoring protocol by the UKB. Then, participants were classified into two groups: (1) Meeting and (2) Not meeting guidelines.

##### 2.2.3.2 Screen‐Based Sedentary Time

We assessed participants' daily total screen‐based ST by combining time spent watching TV and using a computer. Participants were asked, “In a typical day, how many hours do you spend watching TV (using the computer)?” We calculated the daily total ST by combining both TV viewing and computer using time and categorized it into three tertile groups: (1) low (< 3 h/day), (2) moderate (3–4 h/day), and (3) high (> 4 h/day).

##### 2.2.3.3 Sleep Duration

Participants were asked, “About how many hours of sleep do you get every 24 h?”. Based on the National Sleep Foundation's sleep duration recommendations for older adults (7–8 h/day) [21], we classified participants' sleep duration into two groups: (1) Meeting and (2) Not meeting recommendations. For secondary analysis, we also classified participants into three groups based on the duration of sleep: (1) Short (< 7 h/day), (2) Recommended (7–8 h/day), and (3) Long (> 8 h/day).

### 2.3 Covariates

Based on existing literature and knowledge, sociodemographic and lifestyle factors, including age, sex, body mass index, current employment status, average total household income, smoking status, alcohol intake frequencies, and the history of diabetes, cancer, fracture, and other serious medical conditions/disabilities were considered potential confounders on the associations of interest, thereby controlled as covariates in the analysis.

### 2.4 Statistical Analysis

Baseline population characteristics were summarized as frequencies and percentages (*N*;%) for categorical variables and mean with standard deviation (SD) for continuous variables. To assess mean differences between depression groups, we conducted the *chi*‐*square* test of independence for categorical variables and the independent‐sample *t*‐test for continuous variables.

We also measured correlation coefficients between frailty status, frailty indicators, and lifestyle behaviors. Given that all variables were treated as ordered categorical, we utilized the Polychoric correlation coefficient for the assessment.

For investigating the associations of frailty status, frailty indicators, and lifestyle behaviors with depression among older adults, multivariable logistic regression models were applied. First, the likelihood of having depression was predicted with frailty status, frailty indicators, and lifestyle behaviors in the mutually unadjusted model (Model 1). Subsequently, these associations were compared with the fully adjusted model (Models 2 and 3). All models are adjusted by demographic and lifestyle covariates. To further explore the potential moderating role of lifestyle behaviors in the relationship between frailty and depression, interaction terms (i.e., frailty × each lifestyle behavior) were included in the final model. If any significant interaction terms are observed, stratified analyses will be performed. Model fit was assessed through the Hosmer and Lemeshow Goodness‐of‐Fit test, with no evidence indicating a lack of fit (*p* > 0.05), and no multicollinearity issues were found (*r*'s < 0.01). Results from multivariable logistic regression models were presented as odds ratio (OR) with 95% confidence intervals (CI). All statistical analyses were performed using SAS v9.4 (SAS Institute, Cary, NC, USA), with statistical significance at *p* < 0.05.

## 3 Results

### 3.1 Population Characteristics

Of the 69,178 participants aged 65 years or older (mean age = 66.89 ± 1.48 yrs), 20,035 (29%) have depression. A relatively larger portion of older adults with depression are frail (7%) and pre‐frail (45%) when compared to their counterparts without depression (3% and 39% for frail and pre‐frail, respectively). In terms of lifestyle behaviors, depressed older adults have less compliance with MVPA and sleep guidelines and higher total ST (> 4 h) than their nondepressed counterparts [Table 1].

**Table 1:** Comparison of main outcome variables of frailty and lifestyle behaviors across depression statuses.

	Overall	Depression		*p*‐value
		Yes	No	
***N* (%)**	69,178 (100.00)	20,035 (28.96)	49,143 (71.04)	< 0.001*
a **Frailty status**				< 0.001*
Frail	3,048 (4.41)	1,402 (7.00)	1,646 (3.35)	
Pre‐frail	28,373 (41.01)	8,971 (44.78)	19,402 (39.48)	
Non‐frail	37,757 (54.58)	9,662 (48.23)	28,095 (57.17)	
**Frailty indicators**				
**Exhaustion**				< 0.001*
Yes	5,666 (8.19)	2,723 (13.59)	2,943 (5.99)	
No	63,512 (91.81)	17,312 (86.41)	46,200 (94.01)	
**Weight loss**				< 0.001*
Yes	9,717 (14.05)	3,200 (15.97)	6,517 (13.26)	
No	59,461 (85.95)	16,835 (84.03)	42,626 (86.74)	
**Low PA**				< 0.001*
Yes	8,156 (11.79)	2,689 (13.42)	5,467 (11.12)	
No	61,022 (88.21)	17,346 (86.58)	43,676 (88.88)	
**Slow walking speed**				< 0.001*
Yes	7,127 (10.30)	2,654 (13.25)	4,473 (9.10)	
No	62,051 (89.70)	17,381 (86.75)	44,670 (90.90)	
**Low grip strength**				< 0.001*
Yes	14,836 (21.45)	4,863 (24.27)	9,973 (20.29)	
No	54,342 (78.55)	15,172 (75.73)	39,170 (79.71)	
**Lifestyle behaviors**				
**MVPA**				< 0.001*
Meeting	40,769 (58.93)	11,524 (57.52)	29,245 (59.51)	
Not meeting	28,409 (41.07)	8,511 (42.48)	19,898 (40.49)	
**ST**				< 0.001*
Low (< 3 h)	14,411 (20.83)	4,125 (20.59)	10,286 (20.93)	
Moderate (3–4 h)	29,624 (42.82)	8,373 (41.79)	21,251 (43.24)	
High (> 4 h)	25,143 (36.35)	7,537 (37.62)	17,606 (35.83)	
**Sleep**				
Meeting (7–8 h)	46,543 (67.28)	12,560 (62.69)	33,983 (69.15)	< 0.001*
Not meeting (< 7 or > 8 h)	22,635 (32.72)	7,475 (37.31)	15,160 (30.85)	
[Short (< 7 h)]	[14,851 (21.47)]	[4,929 (24.60)]	[9,922 (20.19)]	
[Long (> 8 h)]	[7,784 (11.25)]	[2,546 (12.71)]	[5,238 (10.66)]	

*Note:* Continuous variables were presented as mean with standard deviation, and categorical variables were presented as the frequency (N) and percentage (%).

Abbreviations: BMI, body mass index; MVPA, Moderate‐to‐vigorous physical activity, meeting guidelines (≥ 150 min/wk of moderate or ≥ 75 min/wk of vigorous PA; PA, physical activity; ST, sedentary time; Sleep, meeting guidelines (7 – 8 h/day).

^a^ Frailty status was categorized based on the number of indicators met according to the frailty phenotype criteria: Frail (≥ 3 indicators), Pre‐frail (1‐2 indicators), and Non‐frail (none indicator).

* Significance levels set at *p* < 0.05.

Overall, older adults with depression tend to be female, obese, have low average income, are current/previous smokers, engage in frequent alcohol intake (daily or almost daily), and have a history of being diagnosed with medical conditions or disabilities, compared to their counterparts without depression. Additional population characteristics are presented in [Table 2].

**Table 2:** Descriptive characteristics of baseline population across depression statuses.

	Overall (*N* = 69,178)	Depression		*p*‐value
		Yes (*N* = 20,035)	No (*N* = 49,143)	
**Age (years)**	66.89 (1.48)	66.81 (1.48)	66.93 (1.48)	< 0.001*
**Sex**				< 0.001*
Male	36,745 (53.12)	8,470 (42.28)	28,275 (57.54)	
Female	32,433 (46.88)	11,565 (57.72)	20,868 (42.46)	
**Weight (kg)**	77.56 (14.42)	76.62 (14.52)	77.94 (14.36)	0.006*
**BMI categories (kg/m** ^**2**^ **)**				< 0.001*
< 25	19,713 (29.08)	5,661 (28.90)	14,052 (29.15)	
25– < 30	32,163 (47.45)	9,028 (46.10)	23,135 (47.99)	
≥ 30	15,913 (23.47)	4,896 (25.00)	11,017 (22.85)	
**% Body fat**	31.59 (8.05)	33.19 (8.14)	30.94 (7.92)	< 0.001*
**Ethnicity background**				< 0.001*
British	63,518 (92.08)	18,606 (93.09)	44,912 (91.68)	
Others	5,460 (7.92)	1,382 (6.91)	4,078 (8.32)	
**Qualification**				0.802
College or university degree	17,568 (35.94)	5,051 (35.86)	12,517 (35.98)	
Others	31,308 (64.06)	9,035 (64.14)	22,273 (64.02)	
**Current employment status**				< 0.001*
Retired	58,971 (85.63)	17,288 (86.75)	41,683 (85.18)	
Others	9,894 (14.37)	2,641 (13.25)	7,253 (14.82)	
**Average total household income (£)**				< 0.001*
< 18,000	22,154 (32.02)	7,375 (36.81)	14,779 (30.07)	
18,000–52,000	30,559 (44.17)	8,303 (41.44)	22,256 (45.29)	
> 52,000	5,154 (7.45)	1,117 (5.58)	4,037 (8.21)	
Missing	11,311 (16.5)	3,240 (16.17)	8,071 (16.42)	
**Smoking status**				< 0.001*
Current	4,938 (7.16)	1,610 (8.06)	3,328 (6.80)	
Previous	30,634 (44.45)	9,276 (46.45)	21,358 (43.63)	
Never	33,353 (48.39)	9,085 (45.49)	24,268 (49.57)	
**Alcohol intake frequency**				< 0.001*
Daily or almost daily	14,620 (21.14)	4,825 (24.09)	9,795 (19.94)	
1–4 times/wk	30,932 (44.73)	8,400 (41.94)	22,532 (45.86)	
1–3 times/month or special occasions only	17,571 (25.41)	4,755 (23.74)	12,816 (26.09)	
Never	6,034 (8.73)	2,049 (10.23)	3,985 (8.11)	
**Diabetes diagnosed**				0.137
Yes	5,578 (8.08)	1,663 (8.32)	3,915 (7.98)	
No	63,447 (91.92)	18,318 (91.68)	45,129 (92.02)	
**Cancer diagnosed**				< 0.001*
Yes	8,462 (12.26)	2,656 (13.28)	5,806 (11.84)	
No	60,581 (87.74)	17,339 (86.72)	43,242 (88.16)	
**Fractured/broken bones in last 5 years**				< 0.001*
Yes	6,472 (9.38)	2,219 (11.12)	4,253 (8.68)	
No	62,504 (90.62)	17,735 (88.88)	44,769 (91.32)	
**Other medical conditions or disabilities diagnosed**				< 0.001*
Yes	16,967 (24.88)	5,981 (30.47)	10,986 (22.63)	
No	51,215 (75.12)	13,646 (69.53)	37,569 (77.37)	

*Note:* Continuous variables were presented as mean with standard deviation, and categorical variables were presented as the frequency (N) and percentage (%).

Abbreviations: BMI, body mass index; MET, metabolic equivalent of task; MVPA, moderate‐to‐vigorous physical activity; PA, physical activity.

* Significance levels set at *p* < 0.05.

### 3.2 Correlations Between Frailty and Lifestyle Behaviors

The cross‐tabulations and polychoric correlation matrix between frailty status, frailty indicators, and lifestyle behaviors are presented in [Table 3]. Significant correlations are observed between frailty status, frailty indicators, and lifestyle behaviors (*p*'s < 0.05). Frailty status is negatively correlated with adherence to both MVPA and sleep recommendations (MVPA: *r* = −0.21; Sleep: *r* = −0.16; *p*'s < 0.05). Conversely, a positive correlation was observed with ST (*r* = 0.14; *p* < 0.05). Similar correlation patterns are exhibited between each frailty indicator and lifestyle behavior, except weight loss is positively associated with MVPA (*r* = 0.02; *p* < 0.05).

**Table 3:** Cross‐tabulations and correlation matrix.

	Frailty status			Frailty indicators				
	Frail (*n* = 3,048)	Pre‐frail (*n* = 28,373)	Non‐frail (*n* = 37,757)	Exhaustion (*n* = 5,666)	Weight loss (*n* = 9,717)	Low PA (*n* = 8,156)	Slow walking speed (*n* = 7,127)	Low grip (*n* = 14,836)
a **MVPA (*n*, %)**								
Meeting	929 (30.48)	15,699 (55.33)	24,141 (63.94)	2,678 (47.26)	5,862 (60.33)	2,983 (36.57)	2,583 (36.24)	7,854 (52.94)
Not meeting	2,119 (69.52)	12,674 (44.67)	13,616 (36.06)	2,988 (52.74)	3,855 (39.67)	5,173 (63.43)	4,544 (63.76)	6,982 (47.06)
bCorrelations	−0.206* (1562.55; < 0.001)			−0.159* (347.23; < 0.001)	0.023* (9.07; 0.003)	−0.331* (1909.94; < 0.001)	−0.323* (1690.38; < 0.001)	−0.112* (280.43; < 0.001)
a **ST (*n*, %)**								
Low	406 (13.32)	5,295 (18.66)	8,710 (23.07)	999 (17.63)	1,803 (18.56)	1,301 (15.95)	1,015 (14.24)	2,730 (18.40)
Moderate	1,042 (34.19)	11,715 (41.29)	16,867 (44.67)	2,156 (38.05)	4,043 (41.61)	2,926 (35.88)	2,504 (35.13)	6,123 (41.27)
High	1,600 (52.49)	11,363 (40.05)	12,180 (32.26)	2,511 (44.32)	3,871 (39.84)	3,929 (48.17)	3,608 (50.62)	5,983 (40.33)
bCorrelations	0.139* (835.41; < 0.001)			0.093* (171.05; < 0.001)	0.055* (69.96; < 0.001)	0.153* (565.82; < 0.001)	0.185* (721.07; < 0.001)	0.072* (146.58; < 0.001)
a **Sleep (*n*, %)**								
Meeting	1,441 (47.28)	18,252 (64.33)	26,850 (71.11)	2,833 (50.00)	6,361 (65.46)	4,833 (59.26)	3,859 (54.15)	9,221 (62.15)
Not meeting	1,607 (52.72)	10,121 (35.67)	10,907 (28.89)	2,833 (50.00)	3,356 (34.54)	3,323 (40.74)	3,268 (45.85)	5,615 (37.85)
bCorrelations	−0.164* (121.53; < 0.001)			−0.242* (837.11; < 0.001)	−0.032* (16.96; < 0.001)	−0.128* (270.36; < 0.001)	−0.198* (622.60; < 0.001)	−0.103* (225.53; < 0.001)

Abbreviations: MVPA, moderate‐to‐vigorous pa recommendation adherence, meeting (≥ 150 min/wk of moderate or ≥ 75 min/wk of vigorous PA), ST: low (< 3 h), moderate (3– < 5 h), and high (≥ 5 h), Sleep: Sleep recommendation adherence, meeting (7–8 h/day); PA, physical activity.

^a^ Cross‐tabulation results between frailty and lifestyle behaviors were presented as frequency and percentage.

^b^ Significant correlations were observed between frailty and lifestyle behaviors by Polychoric correlation coefficient. *Chi‐square* test results and *p*‐values were reported with correlation coefficients.

* Significance levels set at *p* < 0.05.

### 3.3 Association Between Frailty Status, Lifestyle Behaviors, and Depression

The results of multivariable logistic regression models for predicting the likelihood of having depression with frailty status and lifestyle behaviors are presented in [Table 4]. Both frailty and pre‐frailty and meeting sleep guidelines are significantly associated with increased odds of depression in older adults, and the statistical significance remains in the fully adjusted model (Model 2; Frail: aOR = 1.87, 95% CI = 1.72, 2.04; Pre‐frail: aOR = 1.22, 95% CI = 1.18, 1.27; Sleep: aOR = 0.81, 95% CI = 0.78, 0.84). Meeting MVPA is significantly associated with depression in the mutually unadjusted model (Model 1; aOR = 0.95, 95% CI = 0.91, 0.98); however, the statistical significance disappears after adjusting for frailty status (Model 2; aOR = 0.99, 95% CI = 0.95, 1.02). Consistently, no significant association is found between ST and depression. No significant interaction effects were observed between lifestyle behaviors and frailty in predicting the likelihood of depression (frailty × MVPA: *Chi*‐square = 1.60, *p* = 0.449; frailty × ST: *chi*‐square = 2.06; *p* = 0.725; frailty × sleep: *Chi*‐square = 0.54, *p* = 0.762).

**Table 4:** Multivariable logistic regression model for predicting the likelihood of having depression with frailty status and lifestyle behaviors.

	Mutually unadjusted	Fully adjusted	
	Model 1^a^	Model 2^b^	Model 3^c^
	OR (95% CI)	OR (95% CI)	OR (95% CI)
**Frailty status**			
Frail	1.96 (1.80, 2.13)*	1.87 (1.72, 2.04)*	—
Pre‐frail	1.24 (1.20, 1.29)*	1.22 (1.18, 1.27)*	—
**Frailty indicators**			
Exhaustion	2.20 (2.08, 2.34)*	—	2.10 (1.97, 2.23)*
Weight loss	1.17 (1.11, 1.23)*	—	1.17 (1.11, 1.23)*
Low PA	1.09 (1.03, 1.15)*	—	0.98 (0.93, 1.04)
Slow walking speed	1.27 (1.20, 1.35)*	—	1.11 (1.04, 1.18)*
Low grip	1.12 (1.07, 1.17)*	—	1.07 (1.02, 1.11)*
**MVPA**			
Meeting	0.95 (0.91, 0.98)*	0.99 (0.95, 1.02)	0.98 (0.95, 1.02)
**ST**			
Moderate	0.97 (0.93, 1.02)	0.97 (0.92, 1.01)	0.97 (0.92, 1.01)
High	1.05 (1.00, 1.10)	1.02 (0.97, 1.07)	1.02 (0.97, 1.07)
**Sleep**			
Meeting	0.79 (0.76, 0.82)*	0.81 (0.78, 0.84)*	0.83 (0.80, 0.86)*

*Note:* References: Non‐frail, Not meeting MVPA and sleep recommendations, low ST (< 3 h). All models are adjusted by covariates of age, sex, BMI, diabetes/cancer/fracture/other medical conditions and disabilities diagnosed, current employment, income, smoking status, and alcohol intake frequencies.

Abbreviations: CI, confidence intervals; MVPA, moderate‐to‐vigorous physical activity recommendation adherence, meeting (≥ 150 min/wk of moderate or ≥ 75 min/wk of vigorous PA); OR, odds ratios; ST, sedentary time, moderate (3– < 5 h), and high (≥ 5 h); Sleep, sleep recommendation adherence, meeting (7–8 h/d).

^a^ Model 1 is mutually unadjusted for each predictor, adjusted for all covariates.

^b^ Model 2 is fully adjusted for frailty status, lifestyle behaviors, and other covariates to examine the independent association between each predictor and depression. Frailty indicators are excluded from this model due to conceptual overlapping with frailty status.

^c^ Model 3 is fully adjusted for frailty indicators, lifestyle behaviors, and other covariates to examine the independent association between each predictor and depression. Frailty status is excluded from this model due to conceptual overlapping with frailty indicators.

* Significance levels set at *p* < 0.05.

### 3.4 Association Between Frailty Indicators, Lifestyle Behaviors, and Depression

All five indicators are significantly associated with higher odds of depression in older adults, and the statistical significance persists in the fully adjusted model (Model 3), except for the low PA indicator (Exhaustion: aOR = 2.10, 95% CI = 1.97, 2.23; Weight loss: aOR = 1.17, 95% CI = 1.11, 1.23; Low PA: aOR = 0.98, 95% CI = 0.93, 1.04; Slow walking speed: aOR = 1.11, 95% CI = 1.04, 1.18; Low grip: aOR = 1.07, 95% CI = 1.02, 1.11). Similar to the frailty status model, the statistical significance of MVPA predicting the likelihood of having depression disappears after adjusting frailty indicators (aOR = 0.98, 95% CI = 0.95, 1.02). Meeting sleep recommendations is consistently associated with lower odds of depression in the fully adjusted model (aOR = 0.83, 95% CI = 0.80, 0.86), while no significant association exists between ST and depression [Table 4].

## 4 Discussion

The current study, encompassing 69,178 older adult participants from the UKB, found that 29% of the participants reported experiencing depression, with more than half belonging to the frail or pre‐frail categories. Importantly, frail or pre‐frail older adults exhibit significantly higher odds of experiencing depression than their non‐frail counterparts. Furthermore, meeting the recommended sleep duration (7–8 h/day) is strongly associated with reduced odds of having depression.

### 4.1 Frailty and Depression in Older Adults

In this study, frail and pre‐frail older adults have 1.87 and 1.22 times higher odds of experiencing depression, respectively, compared to non‐frail older adults. Although there is limited research on frailty as a risk factor for depression, our findings are consistent with existing evidence of a strong independent association between frailty and depression in older adults [3].

One possible explanation is that as frailty is a complex geriatric syndrome influenced by impaired mobility, weakness, and reduced endurance, these risk factors for frailty can contribute to disability, functional dependency, and further development of depression [22]. Other possible mechanisms, such as elevated inflammatory cytokines [23] and dysregulations of hormones associated with frailty [24], may also contribute to developing depression. With recent evidence of their causal relationship through Mendelian randomization study [25], our findings highlight frailty as a predictor of depression in older adults. Given the high prevalence of pre‐frailty and the robust association between frailty as well as pre‐frailty and depression, early prevention or management of frailty could be a potential strategy for addressing depression in older adults.

### 4.2 Physical Activity and Depression

In our study, frailty status is negatively correlated with meeting MVPA recommendations, indicating that meeting MVPA guidelines is associated with lower levels of frailty. Despite the scarcity of robust clinical research examining the effect of PA in managing depression in older adults [26], several studies suggested the benefit of exercise and PA in reducing depression symptoms among older adults [27]. In addition, the evidence supported the positive effects of meeting PA recommendations on attenuating the risk of depression [28]. In our regression Model 1, meeting MVPA guidelines is significantly associated with a 5% reduction in the odds of having depression after adjusting covariates. However, when we extended our analysis by entering variables one by one into the models, we observed that the statistical significance of the relationship between MVPA and depression disappeared after adding frailty status or indicators (Models 2 and 3). Given the significant negative correlation between frailty and MVPA, it indicates a possible mediating effect of frailty on the association between MVPA and depression. However, further investigation is needed to explore the role of frailty as a mediator in this relationship.

### 4.3 Screen‐Based Sedentary Time and Depression

Nevertheless, physical inactivity and being sedentary are known as potential risk factors for both frailty and depression in older adults [12, 16]. In this context, we identify a positive correlation between ST and frailty status, suggesting that increased ST is associated with higher levels of frailty. On the other hand, a significant association between ST and depression is not observed in our study population. Based on previous studies, the relationship between ST and depression varies depending on the type of sedentary behavior and levels of PA, which possibly mitigates the harmful effect of ST on depression [28]. Although we controlled the PA variable in the model, examining the dynamic relationship between PA and ST and its association with depression in older adults is required in future studies with a consideration of the specific type of sedentary behavior and their 24‐h movement behavior construct using objectively measured data.

### 4.4 Sleep Duration and Depression

Meeting sleep guidelines (7–8 h/day) is negatively correlated with frailty and independently associated with approximately 20% lower odds of depression compared to those not meeting sleep guidelines, emphasizing the beneficial effect of adhering to recommended sleep duration. While it was not part of the primary results, we also revealed that both short (< 7 h) and long (> 8 h) sleep duration groups have 1.2 times higher odds of depression than the recommended sleep duration group [Supporting Information: Supplementary Table 2]. Although some previous studies have emphasized the adverse effect of a short duration of sleep on depression [29], our findings are aligned with recent studies, demonstrating that both short and excessive durations of sleep are related to a higher likelihood of having depression in older adults [14, 18]. Several potential mechanisms explain the association between short or long sleep duration and depression, including inflammation [30], disrupted circadian rhythm [31], or stress intolerance [32], which may contribute to an increased risk of depression, but further experimental studies are warranted.

### 4.5 Potential Moderating Effect of Lifestyle Behaviors

Contrary to our initial hypothesis, no significant moderating effects of lifestyle behaviors (i.e., PA, ST, and sleep) were observed in the relationship between frailty and depression among older adults. A possible explanation for this finding is the potential conceptual overlap between the frailty indicator (i.e., low PA component) and lifestyle behaviors, particularly MVPA or ST, where the shared constructs between these variables may influence their potential interaction effect in predicting the odds of depression.

In our study, an adapted version of frailty phenotype criteria incorporates self‐reported PA components, using self‐reported do it yourself (DIY) activities, including light DIY (e.g., pruning, watering the lawn, etc.) and heavy DIY tasks (e.g., weeding, lawn mowing, digging, walking for pleasure, etc.). Physical frailty often reflects a decline in functional capacity to perform daily tasks [33], where DIY activities serve as an appropriate proxy for assessing functional independence in the UKB cohorts. In contrast, self‐reported MVPA variable was derived from IPAQ survey based on the current PA guidelines of 150 min of moderate‐intensity activity or 75 min of vigorous‐intensity activity, which are more indicative of structured PA and exercises associated with broader health benefits [34]. Additionally, time spent in screen‐based sedentary behaviors (i.e., watching TV, using a computer) was used to assess ST to avoid conceptual overlap with other PA variables. Hence, the constructs and implications of different types of PA variables and ST used in our study are distinct, offering complementary perspectives on frailty and lifestyle behaviors. In addition, we confirmed their low correlations and no collinearity issues in the regression models (i.e., *r*'s ≤ 0.1); however, future studies would benefit from investigating their possible overlap in constructs between objectively measured lifestyle behaviors and individual frailty components.

Instead, we further conducted the analysis excluding the frailty PA component from the regression model to explore whether this variable affects the links between other frailty indicators and lifestyle behaviors. The results provide consistent findings, with no significant interaction effects observed [Supporting Information: Supplementary Table 3]. This indicates that the independent associations between frailty, sleep, and depression are robust; still, further longitudinal studies are warranted to examine their dynamic interrelationship changes over time, offering more reliable evidence of the directionality and temporal sequence in these associations.

### 4.6 Strengths and Limitations

The primary strength of this study is that it is a large population‐based study with 69,178 older adults from UKB, investigating the comprehensive relationship between frailty, lifestyle behaviors, and depression in older adults. Additionally, we included PA, ST, and sleep as lifestyle behaviors and assessed their possible moderating role in the links between frailty and depression in older adults. Furthermore, we accounted for various potential confounders, including sociodemographic and lifestyle factors. Consequently, our study findings suggest noteworthy implications for potential nonpharmacological strategies in the management of depression in vulnerable aging populations.

However, it is necessary to acknowledge certain limitations in this study. First, UKB relies on a non‐probability sample methodology, and participants are mostly White British and generally healthy volunteers, leading to selection bias and limiting the generalizability of the findings to general populations. While the large sample size of UKB cohort offers valuable insights into the study findings, future research should validate these findings in more diverse and representative populations to ensure broader applicability. Even though the frailty phenotype has been validated, the self‐reported nature potentially under‐ or overestimates their prevalence and influences reporting bias [2]. Furthermore, the use of the broad definition of depression may result in an overestimated prevalence, as this approach is likely to include individuals with certain personalities or other psychiatric disorders [20]. This raises concern about capturing symptoms beyond typical depressive disorders, potentially inflating prevalence rates. While this approach has been well‐validated in the prior study [20], further research may be needed to cross‐validate our findings across various depression assessment criteria to ensure consistency and generalizability. There may be potentially uncovered overlapping constructs between the frailty PA component and MVPA variable, which could influence the results. Future studies are needed to further explore and address any possible collinearity concerns using objective measures. Although we controlled various potential confounders, additional confounders could impact our findings. Lastly, due to the cross‐sectional study design, a direct causal inference cannot be drawn from our study. Further longitudinal studies are necessary to examine their dynamic interrelationship changes over time, providing more reliable evidence of the directionality and temporal sequence in these associations.

## 5 Conclusion

Frail and pre‐frail older adults are at a greater likelihood of experiencing depression than their non‐frail counterparts. Early intervention targeting frailty and adherence to the recommended sleep duration (7–8 h/day) can be used as a modifiable behavioral strategy to address or mitigate depression in older adults. Further longitudinal research utilizing objectively measured data is imperative to systematically examine this complex interconnected association changes over time, to offer stronger evidence of directionality in these associations.

## Author Contributions

**Jisu Kim:** data curation, investigation, formal analysis, writing – original draft, methodology. **Jonathan Kenyon:** investigation, methodology, writing – review and editing. **Juan Lu:** methodology, writing – review and editing. **Lana Sargent:** writing – review and editing. **Youngdeok Kim:** data curation, investigation, formal analysis, methodology, writing – review and editing, project administration.

## Ethics Statement

The UKB project was approved by the North West Multicenter Research Ethical Committee (11/NW/0382).

## Conflicts of Interest

The authors declare no conflicts of interest.

## Transparency Statement

The lead author (J.S.K) affirms that this manuscript is an honest, accurate, and transparent account of the study being reported; that no important aspects of the study have been omitted; and that any discrepancies from the study as planned (and, if relevant, registered) have been explained.

## Supporting information

Supporting information.

## Data Availability

The data that support the findings of this study are available in UK Biobank at https://www.ukbiobank.ac.uk/. These data were derived from the following resources available in the public domain: ‐ UK Biobank, https://www.ukbiobank.ac.uk/. The corresponding author (Y.D.K) had full access to the data used in this study and takes complete responsibility for the integrity of the data and the accuracy of the data analysis. Detailed information for these publicly available datasets used in the study can be found in the UKB website (https://www.ukbiobank.ac.uk/).
