# Supplementary material for: Among 69,178 UK Residents Ages 65+ Years, Frailty Associates Significantly With Lifestyle Behaviors and Depression: A Cross‐Sectional Study
Source: Health Sci Rep. 2025 Mar 26;8(3):e70593. doi: 10.1002/hsr2.70593 (PMC11938289; doi:10.1002/hsr2.70593)
Supplement: Supplementary file 1 — Supporting information. [file HSR2-8-e70593-s001.docx]

| **Supplementary Table 1. Frailty phenotype criteria in the UK Biobank.** | | |
| --- | --- | --- |
| **Indicators** | **Descriptions** | **Categories** |
| Exhaustion | Self-reported: “Over the past two weeks, how often have you felt tired or had little energy?” | 1: More than half the days or nearly every day  0: Others |
| Weight loss | Self-reported: “Compared with one year ago, has your weight changed?” | 1: Yes – lost weight  0: Others |
| Low physical activity | Self-reported using UKB physical activity questionnaire: “In the last 4 weeks, did you spend any time doing the following?” The responses were categorized into:   - None (No physical activity) - Low (light DIY^a^ activity – e.g., pruning, watering the lawn) - Medium (heavy DIY activity – e.g., weeding, lawn mowing, carpentry, and digging; walking for pleasure, or other exercises) - High (strenuous sports) | 1: None or light DIY with a frequency of once per week or less  0: Others |
| Slow walking speed | Self-reported: “How would you describe your walking pace?” | 1: Slow  0: Others |
| Low grip strength | Measured grip strength (sex and BMI were adjusted using cutoffs from Fried et al. [1]) | 1:  (1) Men: ≤29 kg for BMI ≤24 kg/m^2^; ≤30 kg for BMI 24.1-26 kg/m^2^; ≤30 kg for BMI 26.1-28 kg/m^2^; or ≤32 kg for BMI >28 kg/m^2^  (2) Women: ≤17 kg for BMI ≤23 kg/m^2^; ≤17.3 kg for BMI 23.1-26 kg/m^2^; ≤18 kg for BMI 26.1-29 kg/m^2^; or ≤21 kg for BMI >29 kg/m^2^  0: Others |
| ^a^DIY: Do It Yourself; ^b^BMI: Body-Mass Index | | |

| **Supplementary Table 2. Multivariate logistic regression model for predicting the likelihood of having depression with frailty status and lifestyle behaviors, including sleep duration.** | | | | | | | | | | | | |
| --- | --- | --- | --- | --- | --- | --- | --- | --- | --- | --- | --- | --- |
|  | **Mutually unadjusted, adjusted for covariates** | | | | | | **Fully adjusted** | | | | | |
|  | **Model 1-1** | | | | | | **Model 1-2** | | | | | |
|  | ***b*** | **SE** | **OR** | **95% CI** | | ***p*-value** | ***b*** | **SE** | **OR** | **95% CI** | | ***p*-value** |
|  |  |  |  | **LL** | **UL** |  |  |  |  | **LL** | **UL** |  |
| **Frailty status** |  |  |  |  |  |  |  |  |  |  |  |  |
| Frail | 0.671 | 0.043 | 1.957 | 1.799 | 2.128 | <.0001* | 0.630 | 0.044 | 1.878 | 1.724 | 2.045 | <.0001* |
| Pre-frail | 0.215 | 0.019 | 1.240 | 1.195 | 1.286 | <.0001* | 0.204 | 0.019 | 1.226 | 1.182 | 1.271 | <.0001* |
| **MVPA** |  |  |  |  |  |  |  |  |  |  |  |  |
| meeting | -0.056 | 0.018 | 0.946 | 0.913 | 0.980 | 0.002* | -0.013 | 0.018 | 0.987 | 0.952 | 1.023 | 0.466 |
| **ST** |  |  |  |  |  |  |  |  |  |  |  |  |
| moderate | -0.030 | 0.024 | 0.970 | 0.926 | 1.016 | 0.203 | -0.035 | 0.024 | 0.966 | 0.922 | 1.012 | 0.145 |
| high | 0.047 | 0.025 | 1.049 | 0.999 | 1.101 | 0.057 | 0.021 | 0.025 | 1.021 | 0.972 | 1.073 | 0.399 |
| **Sleep** |  |  |  |  |  |  |  |  |  |  |  |  |
| < 7h | 0.227 | 0.021 | 1.255 | 1.203 | 1.308 | <.0001* | 0.204 | 0.022 | 1.226 | 1.178 | 1.279 | <.0001* |
| >8 h | 0.253 | 0.028 | 1.287 | 1.219 | 1.359 | <.0001* | 0.221 | 0.028 | 1.248 | 1.181 | 1.318 | <.0001* |
| References: non-frail, not meeting MVPA and sleep recommendation, low ST (<3h).  Abbreviations: SE: Standard Errors; OR: Odds Ratios; CI: Confidence Intervals; LL: Lower Levels; UL: Upper Levels; MVPA: Moderate-to-Vigorous Physical Activity recommendation adherence, meeting (≥150 mins/wk of moderate or ≥ 75 mins/wk of vigorous PA); ST: Sedentary Time, moderate (3-<5h), and high (≥5h); Sleep: Sleep recommendation adherence (reference: 7 – 8h). Adjusted by covariates of age, sex, BMI, diabetes/cancer/fracture/other medical conditions and disabilities diagnosed, current employment, income, smoking status, and alcohol intake frequencies.; Significance levels are set at *p*<.05*. | | | | | | | | | | | | |

| **Supplementary Table 3**. Multivariate logistic regression model for predicting the likelihood of having depression with frailty indicators, excluding a low physical activity indicator, and lifestyle behaviors | | | | | | |
| --- | --- | --- | --- | --- | --- | --- |
|  | **Fully adjusted** | | | | | |
|  | **Model** | | | | | |
|  | ***b*** | **SE** | **OR** | **95% CI** | | ***p*-value** |
|  |  |  |  | **LL** | **UL** |  |
| **Frailty indicators** |  |  |  |  |  |  |
| Exhaustion | 0.739 | 0.031 | 2.093 | 1.970 | 2.223 | <.0001* |
| Weight loss | 0.156 | 0.025 | 1.169 | 1.113 | 1.227 | <.0001* |
| Slow walking speed | 0.100 | 0.031 | 1.105 | 1.040 | 1.173 | 0.001* |
| Low grip | 0.064 | 0.022 | 1.066 | 1.021 | 1.113 | 0.004* |
| **MVPA** |  |  |  |  |  |  |
| meeting | -0.016 | 0.018 | 0.984 | 0.950 | 1.020 | 0.389 |
| **ST** |  |  |  |  |  |  |
| moderate | -0.033 | 0.023 | 0.968 | 0.923 | 1.014 | 0.167 |
| high | 0.020 | 0.025 | 1.020 | 0.971 | 1.072 | 0.426 |
| **Sleep** |  |  |  |  |  |  |
| meeting | -0.188 | 0.019 | 0.829 | 0.799 | 0.860 | <.0001* |
| Reference: non-frail, not meeting MVPA and sleep recommendation, low ST (<3h).  The low physical activity variable was excluded due to conceptual overlap with MVPA.  SE: Standard Errors; OR: Odds Ratios; CI: Confidence Intervals; LL: Lower Levels; UL: Upper Levels; MVPA: Moderate-to-Vigorous Physical Activity recommendation adherence, meeting (≥150 mins/wk of moderate or ≥ 75 mins/wk of vigorous PA); ST: Sedentary Time, moderate (3-<5h), and high (≥5h); Sleep: Sleep recommendation adherence, meeting (7 – 8h/day). The model is adjusted by covariates of age, sex, BMI, diabetes/cancer/fracture/other medical conditions and disabilities diagnosed, current employment, income, smoking status, and alcohol intake frequencies. | | | | | | |
